# *Aegilops tauschii* Genome Sequence: A Framework for Meta-analysis of Wheat QTLs

**DOI:** 10.1534/g3.118.200921

**Published:** 2019-01-22

**Authors:** Jiale Xu, Xiongtao Dai, Ramesh K. Ramasamy, Le Wang, Tingting Zhu, Patrick E. McGuire, Chad M. Jorgensen, Hamid Dehghani, Patrick J. Gulick, Ming-Cheng Luo, Hans-Georg Müller, Jan Dvorak

**Affiliations:** * Department of Plant Sciences, University of California, Davis, California,; ** Department of Statistics, University of California, Davis, California,; † Department of Statistics, Iowa State University, Iowa,; ‡ Department of Plant Breeding, Faculty of Agriculture, Tarbiat Modares University, Tehran, Iran, and; § Department of Biology, Concordia University, Montreal, Quebec, Canada

**Keywords:** QTL distribution, meta-QTL, QTL database, probability, Web portal, recombination rate, fusarium head blight

## Abstract

Numerous quantitative trait loci (QTL) have been mapped in tetraploid and hexaploid wheat and wheat relatives, mostly with simple sequence repeat (SSR) or single nucleotide polymorphism (SNP) markers. To conduct meta-analysis of QTL requires projecting them onto a common genomic framework, either a consensus genetic map or genomic sequence. The latter strategy is pursued here. Of 774 QTL mapped in wheat and wheat relatives found in the literature, 585 (75.6%) were successfully projected onto the *Aegilops tauschii* pseudomolecules. QTL mapped with SNP markers were more successfully projected (92.2%) than those mapped with SSR markers (66.2%). The QTL were not distributed homogeneously along chromosome arms. Their frequencies increased in the proximal-to-distal direction but declined in the most distal regions and were weakly correlated with recombination rates along the chromosome arms. Databases for projected SSR markers and QTL were constructed and incorporated into the *Ae. tauschii* JBrowse. To facilitate meta-QTL analysis, eight clusters of QTL were used to estimate standard deviations ($\hat{\sigma }$) of independently mapped QTL projected onto the *Ae. tauschii* genome sequence. The standard deviations $\hat{\sigma }$ were modeled as an exponential decay function of recombination rates along the *Ae. tauschii* chromosomes. We implemented four hypothesis tests for determining the membership of query QTL. The hypothesis tests and estimation procedure for $\hat{\sigma }$ were implemented in a web portal for meta-analysis of projected QTL. Twenty-one QTL for *Fusarium* head blight resistance mapped on wheat chromosomes 3A, 3B, and 3D were analyzed to illustrate the use of the portal for meta-QTL analyses.

Wheat is the most widely grown field crop and an important source of protein and carbohydrates in the human diet. Intelligent manipulation of genetic variation of economically important traits in wheat, most of them quantitative, is essential for sustaining wheat genetic improvement and meeting demands for wheat grain in the future.

Commercially important wheats are hexaploid bread wheat (*Triticum aestivum*, subgenomes BBAADD) and tetraploid durum wheat (*T. turgidum* ssp. *durum*, subgenomes BBAA). Wheat subgenomes were contributed by *T. urartu* (A subgenome), a close relative of *Aegilops speltoides* (B subgenome), and *Ae. tauschii* (D subgenome) (McFadden and Sears 1946; Dvorak and Zhang 1990; Dvorak *et al.* 1993). The durum A and B subgenomes, as well as those of wild tetraploid emmer wheat (*T. turgidum* ssp. *dicoccoides*), are equivalent to the A and B subgenomes of bread wheat.

The bread wheat genome consists of 21 chromosomes. They were allocated into seven groups of three homeologous chromosomes, one from each subgenome (Sears 1966). The ability of the three chromosomes within each homeologous group to replace each other in the nullisomic-tetrasomic aneuploid lines indicated that the majority of wheat orthologous genes have the same or similar functions. The same is true for genomes of most other species in the tribe Triticeae, as compensation in the numerous disomic substitution lines that have been developed in the wheat genetic background has revealed. In addition to wheat, the tribe includes other cereals, such as barley and rye, and nearly 300 grass species (Löve 1984). Most of them can be hybridized with wheat and represent an important genetic resource for wheat improvement. Although wheat cannot be sexually hybridized with grasses outside of the tribe, they are nevertheless important for wheat genomics (Lin *et al.* 1995). The functions of genes in other grass genomes suggest functions of wheat genes and facilitate the discovery of candidate genes for wheat qualitative and quantitative traits.

The importance of genome comparisons for wheat genetics and breeding became evident at the onset of wheat molecular mapping (Gale and Devos 1998), which was based on restriction fragment length polymorphism (RFLP) markers. RFLP markers had numerous drawbacks but were comparative. Subsequently, more than 8,000 simple sequence repeat (SSR) markers have been developed in wheat (https://wheat.pw.usda.gov/GG3/). The primers for most of them were engineered to be subgenome specific to facilitate mapping at the polyploid level. Wheat SSR markers are therefore poorly suited for comparative mapping. The use of single nucleotide polymorphism (SNP) markers is currently gaining importance because of their abundance in wheat and wheat relatives and their suitability for genotyping automation (Akhunov *et al.* 2009; Luo *et al.* 2009; Akhunov *et al.* 2010; Luo *et al.* 2013; Wang *et al.* 2014). Gene-based SNP markers can be detected by homology searches in related genomes and are therefore more comparative than SSR markers.

Many genetic maps have been constructed with RFLP, SSR, and SNP markers and some have been integrated into consensus maps (Somers *et al.* 2004; Marone *et al.* 2012; Maccaferri *et al.* 2015). Consensus genetic maps create a framework for integration of quantitative trait loci (QTL) from different maps and their meta-QTL analyses (Quraishi *et al.* 2017). A meta-QTL analysis is a method derived from meta-analysis, a statistical concept of integrating independently obtained data into a single analysis (Goffinet and Gerber 2000). Major assets of meta-QTL analyses built from independent QTL studies (Veyrieras *et al.* 2007) include increased QTL detection power, reduced size of confidence interval in which a QTL is located, and better control of false positive candidate-gene discovery. Meta-QTL analysis has been successfully deployed in wheat (Hanocq *et al.* 2007; Griffiths *et al.* 2009; Tyagi *et al.* 2015). A consensus genetic map involving 140,000 molecular markers hosting 32 meta-QTL has been built by combining four wheat genetic maps and 27 wheat QTL studies (Quraishi *et al.* 2017).

The backbone for integrating markers, Mendelian genes, and QTL should ideally be a genome sequence, not a genetic map (Jia *et al.* 2013). A genome sequence is comparative and markers can be incorporated into it on a large scale by searches for homology. QTL can be projected onto a genome sequence relative to each other and relative to genes annotated in the genome sequence, thus streamlining candidate-gene discovery.

This strategy for meta-QTL analysis in Triticeae has been until recently largely hypothetical because no reference-quality genome sequence was available in the tribe. The publication of genome sequences for both wild ancestors of wheat, the tetraploid wild emmer wheat (Avni *et al.* 2017) and *Ae. tauschii* (Luo *et al.* 2017; Zhao *et al.* 2017) has changed this situation. *Ae. tauschii* genome sequence Aet v4.0 (Luo *et al*. 2017) has been used as a backbone for large-scale projections of markers from a genetic map onto the genome sequence (Jorgensen *et al*. 2017). Of 10,914 A- and B-subgenome SNP markers mapped on an ultra-dense genetic map of wild emmer wheat, 9,131 (83.6%) were successfully projected onto the *Ae. tauschii* Aet v4.0 pseudomolecules (Jorgensen *et al.* 2017) providing proof of concept. Zhao *et al.* (Zhao *et al.* 2017) later also reported success with projecting molecular markers and QTL onto another *Ae. tauschii* genome sequence.

Based on our success with projecting SNP markers onto the Aet v4.0 genome sequence, we evaluated here the projection of wheat SSR markers onto the *Ae. tauschii* pseudomolecules. We then used projected markers to project QTL onto the *Ae. tauschii* pseudomolecules. We performed a meta-analysis of eight clusters of projected QTL clustering about their causative Mendelian loci to estimate standard deviations of mapping and projecting QTL onto the *Ae. tauschii* genome sequence. We observed that the size of standard deviation decayed exponentially as the meiotic recombination rate along the *Ae. tauschii* chromosome arms (Luo *et al.* 2017) increased and derived a model for computing standard deviation of projected QTL onto the *Ae. tauschii* Aet v4.0 pseudomolecules. Three hypothesis tests were proposed to investigate the association of a new (query) QTL with an existing individual QTL (QTL *vs.* QTL), a Mendelian gene (QTL *vs.* Mendelian locus), or known cluster of QTL (QTL *vs.* cluster mean). A fourth test for detecting multiple clusters within a set of QTL was constructed from the QTL-vs-QTL test. Finally, we developed a portal for computing standard deviations adjusted for local recombination rates and probabilities for meta-QTL analyses and performed a case study for 21 QTL for *Fusarium* head blight resistance mapped on the three chromosomes of homeologous group 3 to illustrate the use of the portal and hypothesis testing.

## Methods

### QTL data retrieval

Data for 258 SNP-associated QTL and 500 SSR-associated QTL were extracted from 97 studies in wheat and wheat relatives. In addition, 16 additional QTL mapped with some other marker types, *e.g.*, EST, were also collected. The QTL were for traits related to biotic stress resistance, abiotic stress tolerance, grain yield, grain quality, and various physiological and morphological traits. For QTL mapped relative to a single marker, nucleotide sequence of the marker was retrieved from the relevant marker database. For QTL mapped relative to two flanking markers, sequences for both flanking markers were retrieved from the database.

Of the 258 QTL mapped with SNP markers, 226 were mapped with an SNP array, such as the 90K Illumina iSelect Wheat Infinium SNP array (Wang *et al.* 2014), 9K iSelect Wheat BeadChips array (Cavanagh *et al.* 2013), and the 35K Wheat Breeder’s Array (Allen *et al.* 2017). The remaining 32 QTL were mapped by genotyping by sequencing (GBS) (Zhou *et al.* 2017). Sequences of the 90K and 9K SNP markers were downloaded from the Kansas University SNP marker database (http://wheatgenomics.plantpath.ksu.edu/snp/) and those of the 35K SNP markers were downloaded from the University of Bristol CerealsDB (http://www.cerealsdb.uk.net/cerealgenomics/CerealsDB/indexNEW.php). For the 32 GBS markers, sequences of the relevant reads were retrieved from the Chromosome Survey Sequence published by IWGSC (https://www.wheatgenome.org/). Primer sequences of 6,385 SSRs were downloaded from the GrainGenes database (https://wheat.pw.usda.gov/GG3/). In addition to marker sequences, positions on a genetic map, mapping population, and journal reference were recorded.

### Projection of wheat SNP and SSR markers onto the Ae. tauschii genome sequence

SNP marker sequences and GBS reads were used as queries against the *Ae. tauschii* reference genome sequence Aet v4.0 (Luo *et al.* 2017). Homology searches were performed (https://www.cyverse.org/acknowledge-and-cite-cyverse) with BLASTN in the NCBI BLAST+ v2.7.1 package using the default search parameters except for word-size, which was adjusted to 8. A hit was considered successful if it was located on the *Ae. tauschii* chromosome homeologous to the wheat chromosome on which the SNP or GBS marker was originally mapped. Hits on other *Ae. tauschii* chromosomes or multiple hits were discarded. If there was more than a single hit on the *Ae. tauschii* homeologue, the SNP marker was discarded.

Because SSR primers are short, BLASTN homology search with SSR primers as queries was first performed against the *Triticum aestivum* cv Chinese Spring (CS) pseudomolecules (http://aegilops.wheat.ucdavis.edu/ATGSP/blast.php), to retrieve the entire amplicon. The hits were filtered as follows. (1) A hit had at least a 95% identity. (2) The distance between the forward and reverse primer sequences was < 1 kb (changing this filter to < 10 kb was explored and is reported). (3) The hit was located on the wheat pseudomolecule corresponding to the location of the SSR marker in the GrainGenes database. The sequence of the amplicon was then used as a query in a BLASTN homology search against the *Ae. tauschii* genome sequence. If the search resulted in hits on several *Ae. tauschii* pseudomolecules the hit on the homeologous pseudomolecule was selected. If there was more than a single hit on the *Ae. tauschii* homeologues, the marker was discarded.

### Projecting and validating QTL on the Ae. tauschii genome sequence

A QTL was projected onto an *Ae. tauschii* pseudomolecule Aet v4.0 by projecting either a single marker near the LOD score peak or a pair of flanking markers associated with the QTL LOD score. If a pair of markers was projected, the QTL was assumed to be located at the midpoint between the two marker locations. To validate a QTL projection based on a single marker, another marker in the proximity of the projecting marker was selected from the linkage map in the original study or from a genetic map in the GrainGenes database. This additional marker was projected onto the *Ae. tauschii* genome sequence. If the projection of the additional marker was in the vicinity of the original marker, this additional marker was considered to be a validating marker and the QTL location was considered validated. For QTL projected onto the sequence by a pair of flanking markers, a QTL was considered validated if both markers were located in the *Ae. tauschii* genome sequence in the vicinity of each other. If one of the two flanking markers could not be projected onto the sequence, another marker was selected and used for validating the QTL as described above.

### Distribution of projected QTL

In the *Ae. tauschii* genome, gene density increases about fourfold from the centromere toward the telomere (Luo *et al.* 2017). To assess the distribution of QTL along chromosome arms, the numbers of QTL were expressed per bin containing equal numbers of genes rather than per Mb. Genes in an arm were divided into five bins, each containing 20% of genes in the arm ordered along the centromere-telomere axis. The frequency of QTL (the number of QTL per bin) was computed for each bin. Frequencies of QTL across different chromosome arms were therefore comparable and could be averaged.

The analysis of QTL distribution was performed separately for the long arms and short arms. In addition, we analyzed the effects of marker (SSR or SNP) used for QTL mapping on the QTL distribution along the long and short arms.

### JBrowse tracks and QTL naming

Databases containing projected markers and QTL were included into the *Ae. tauschii* Aet v4.0 genome sequence JBrowse database (http://aegilops.wheat.ucdavis.edu/jbrowse/index.html?data=Aet/data). Marker and QTL tracks were added to the JBrowse graphical display (File S1). To incorporate projected markers and QTL into the JBrowse, each QTL received an identification number (ID). ID starts with the letter “Q” followed by a four-digit number. The first digit indicates the *Ae. tauschii* pseudomolecule (1D to 7D). The second digit indicates a 100 Mb section starting at the tip of the short arm of the *Ae. tauschii* pseudomolecule. The third digit places QTL into ten sections and the last digit subdivides each section into 10 sub-sections, starting at the midpoint. Therefore, a 100 Mb section can contain up to 100 ordered QTL. QTL that are sharing the same four digits because of the same associated marker are differentially designated by using one or two digits after a decimal point. For example, for QTL Q1028.1, the first digit indicates that the QTL was on pseudomolecule 1D, the second digit indicates that it was in the section 0-100 Mb from the tip of the short arm, the third and fourth digits (2 and 8) indicate that the QTL was arbitrarily placed in the third sub-section and ninth sub-sub-section of the 100-Mb section. The decimal digit (0.1) indicates that there are several QTL, which are projected at the same location of pseudomolecule 1D.

### Meta-QTL hypothesis testing

Testing meta-QTL hypotheses requires an estimate of the standard deviation of independent QTL mapping attempts. To estimate the standard deviation, eight clusters of QTL for which the underlying Mendelian loci were known were constructed.

***GW2* cluster** *GW2* encodes E3 ubiquitin-protein ligase and regulates grain weight in rice (Kikuchi *et al.* 2003). The wheat ortholog *GW2* on 6A negatively regulates grain width and thousand kernel weight (TKW) (Song *et al.* 2007). Three A- and B-genome QTL affecting TKW (Huang *et al.* 2006; Wu *et al.* 2015; Gao *et al.* 2015) projected in the proximity of *GW2* were used as a *GW2* cluster.
***Glu-1* cluster** *Glu-1* encodes high-molecular-weight glutenin subunits (HMW-GS) (Forde *et al.* 1985). The locus is proximally located in the long arms of wheat chromosomes 1A, 1B, and 1D and is a major determinant of wheat baking quality (Payne *et al.* 1981). Ten A- and B-genome QTL for various measures of wheat baking quality, such as falling number (FN), test weight (TW), grain protein content (GPC), mixograph peak height (PH), sedimentation volume (SED), and dough strength (DS), known to be associated with *Glu-1* were used as the *Glu-1* cluster.
***GLEN42K* cluster** *GLEN42K* is a member of a multigene locus *Glu-3* located in the distal ends of chromosomes 1A, 1B, and 1D and encodes a LMW-s (serine) subunit, one of the components of low-molecular-weight glutenin (LWM-GS) (Maruyama-Funatsuki *et al.* 2005). This subunit is thought to be associated with good pasta-making quality (Pogna *et al.* 1988;Masci *et al.* 2000). Five A-, B-, and D-genome QTL (McCartney *et al.* 2005; Arbelbide and Bernardo 2006; Yu *et al.* 2017; Zou *et al.* 2017) for several measures of wheat baking quality, such as SED, TW, and grain hardness (GH), were used as this cluster.
***Vrn-B1* cluster** *Vrn-B1* encodes MADS-box protein TaVRT-1 (Yan *et al.* 2003), and deletions within this gene are associated with altered vernalization requirements and spring growth habit in wheat (Fu *et al.* 2005). Four A- and B-genome QTL (Hanocq *et al.* 2004; Hanocq *et al.* 2007) for vernalization requirement (VR) projected in the proximity of *Vrn-1* were used as the *Vrn-1* cluster.
***Yr10* cluster** *Yr10* encodes a CC-NBS-LRR sequence in wheat (Liu *et al.* 2014) that confers stripe rust resistance (SRR) in wheat cultivars (*e.g.*, Moro) and was located at the tip of the short arm of chromosome 1B (Metzger and Silbaugh 1970). Three B- and D-genome SRR QTL (Hou *et al.* 2015; Naruoka *et al.* 2015) projected in the proximity of *Yr10* were used as the *Yr10* cluster.
***HKT4* cluster** *HKT4* encodes cation transporter that mediates potassium (K^+^) uptake in rice (Garciadeblás *et al.* 2003). Comparative mapping in rice, barley and wheat revealed that the wheat ortholog was on the long arm of chromosome 2B (Huang *et al.* 2008). Two QTL (Lindsay *et al.* 2004; Hussain *et al.* 2017) for shoot K^+^ concentration (SKC) were projected in the proximity of *HKT4* gene and were used as the *HKT4* cluster.
***Ppd-1* cluster** *Ppd-1* encodes a key floral regulator. The gene was located on wheat chromosomes 2A, 2B, and 2D (Worland 2001;Beales *et al.* 2007;Wilhelm *et al.* 2009). Its mutations confer photoperiod insensitivity. Six QTL (Huang *et al.* 2003; Xu *et al.* 2005; Huang *et al.* 2006; Kunert *et al.* 2007) projected in the proximity of *Pdp-1* and affecting photoperiod sensitivity and several other related traits, such as heading date (HD), days to maturity (DTM), ear emergence time (EET), and flowering time (FT), were used as the *Ppd-1* cluster.
***Rht-1* cluster** *Rht-1* encodes in rice the DELLA protein and acts as a repressor of gibberellin (GA) responsive growth (Gale and Marshall 1973; Pinthus *et al.* 1989). Wheat orthologs *Rht-B1 and Rht-D1* were mapped on the short arms of 4BS and 4DS (Sourdille *et al.* 1998). Allelic variants of these two genes result in dwarfing with a broad range of plant height in wheat (Gale *et al.* 1985;Börner *et al.* 1996). Three QTL (McCartney *et al.* 2005; Gao *et al.* 2015) affecting plant height (PTH) and projected in the proximity of *Rht-1* were used as the *Rht-1* cluster.

Each QTL was projected onto the *Ae. tauschii* genome sequence, and pairwise distances of QTL associated with the same Mendelian locus were used to estimate the standard deviation of a QTL cluster. Because distances between markers on Triticeae genetic maps are dependent on recombination rates along chromosomes, the local recombination rate must be taken into account in computing the *p*-values in meta-QTL hypothesis testing. To enumerate the local recombination rate, each pseudomolecule was divided by 10,000 equally spaced grid points. Based on the *Ae. tauschii* genetic map (Luo *et al.* 2013), the recombination rate at each grid point was computed by applying local cubic smoothing with Gaussian kernel and 5-Mb bandwidth (Luo *et al.* 2013; Dai *et al.* 2018).

Within a cluster, the standard deviation σ of the distance between the projected QTL location and the mean location of QTL mapping attempts for the same Mendelian locus was estimated by a robust scale estimator (Rousseeuw and Croux 1993) $\hat{\sigma }=2.2219\times \text{first quartile of}(\left|x_{i}-x_{j}\right|:i<j)$. The average recombination rate (Rt) was estimated by using recombination rates of the grid points in the cluster. A preliminary exploratory analysis found that the relationship between $\hat{\sigma }$ and rate Rt was properly described by an exponential decay. This suggested the use of the linear regression model

$$\log\left(\hat{\sigma }\right)=\text{α}+\beta \text{Rt}+\epsilon$$

using the logarithm of the standard deviation estimate $\hat{\sigma }$ as response and Rt in the chromosome region spanning the QTL cluster as predictor. After obtaining least square estimates $\hat{\alpha }$ and $\hat{\beta }$, the regression model was exponentiated and $\tilde{\sigma }=\text{exp}\left(\hat{\alpha }+\hat{\beta }\text{Rt}\right)$ was used for computing the recombination-dependent standard deviation within a cluster.

To compare dispersion of QTL mapped with SSR markers with those mapped with SNP markers, the adjusted distance of each QTL to the mean location of QTL in a cluster was computed as

$$D_{ij}=\frac{\left|X_{ij}-{\bar{X}}_{j}\right|\;}{{\tilde{\sigma }}_{j}},$$

where $D_{ij}$ is the adjusted distance of the *i*th QTL in the *j*th cluster, ${\bar{X}}_{j}\;$is the mean location of QTL in the *j*th cluster, and ${\tilde{\sigma }}_{j}$ is the standard deviation of the *j*th cluster predicted by the regression model. QTL were then divided into two groups based on whether mapping was based on SSR markers or on SNP markers. A *t*-test based on the adjusted distances was performed for the null hypothesis *H_0_*: the mean adjusted deviations of QTL mapped by two different marker types are the same, which would hold if the size of standard deviation were not affected by marker type.

### Model applications

The following methods can be used to assess whether a new (query) QTL belongs to the same cluster as a single known QTL (Test 1), a Mendelian gene (Test 2), or a set of QTL forming a known cluster (Test 3). A Mendelian gene in Test 2 is a gene of known location in the *Ae. tauchii* genome sequence. A known cluster in Test 3 is a group of QTL for which there is evidence that they are independent estimates of a single Mendelian locus of unknown location in the *Ae. tauschii* genome sequence. We consider the following hypothesis tests for these scenarios:

Test 1: $H_{0}$: X and Y are QTL of the same gene *vs.* $H_{1}$:$\;H_{0}$ is not true,
Test 2: $H_{0}$: X is associated with a Mendelian gene of known location in the genome sequence *vs.* $H_{1}$:$\;H_{0}$ is not true,
Test 3: $H_{0}$: X and $\left(Y_{1},\ldots ,Y_{m}\right)$ are QTL of the same Mendelian gene *vs.* $H_{1}$:$\;H_{0}$ is not true,

where the location (in Mb) of the new QTL is denoted as X, that of the known (respectively set of) QTL as Y (respectively $Y_{1},\ldots ,Y_{n})$, and the mean location (in Mb) of the QTL cluster associated with underlying Mendelian locus corresponding to the new QTL X as $\mu_{1}$ and that of the known QTL(s) as $\;\mu_{2}$, respectively. The null hypotheses of interest can then be written as

$$\;H_{0}:\mu_{1}=\mu_{2}.$$

Assuming $X∼N\left(\mu_{1},\sigma^{2}\right)$, and $Y,Y_{1},\ldots ,Y_{n}∼N(\mu_{2},\sigma^{2})$, respectively, where σ is the standard deviation of the locations of the QTL associated with the same gene, we used the *z*-test to obtain the *p*-value for testing $H_{0}$ against $H_{1}$. The test statistic T and two-sided *p*-value for each test were

$\text{Test }1:T=X-Y∼N\left(0,2\sigma^{2}\right)$, $p=2(1-\Phi (|X-Y|/(\sqrt{2}\;\sigma )))$,
Test 2: $T=X-\mu_{2}∼N\left(0,\sigma^{2}\right)$, $p=2\left(1-\Phi \left(\left|X-\mu_{2}\right|/\sigma \right)\right)$.
$\text{Test }3:T=X-{\bar{Y}}_{m}∼N\left(0,(1+m^{-1})\sigma^{2}\right)$, $p=2\left(1-\Phi \left(\left|X-Y\right|/(\sqrt{\left(1+m^{-1}\right)}\sigma )\right)\right)$.

The standard deviation σ was estimated by $\tilde{\sigma }=\text{exp}(\hat{\alpha }+\hat{\beta }\text{Rt})$ where Rt is the average recombination rate of the region spanning the QTL cluster. We assume that this estimation procedure is consistent, which then justifies the application of the *z*-test for large sample sizes.

Consecutive applications of Test 1 can also assess whether a given set of QTL at location $X_{1},\ldots ,X_{n}$ form a single cluster. Specifically, to test the hypothesis

Test 4: $H_{0}$:$\;X_{1},\ldots ,X_{n}$ are QTL of the same gene *vs.* $H_{1}$:$\;H_{0}$ is not true,

one can apply Test 1 on neighboring QTL pairs ${\left\{\left(X_{\left[i\right]},X_{\left[i+1\right]}\right)\right\}}_{i=1}^{n-1}$, where $X_{\left[i\right]}$ is the *i*th smallest QTL location, and reject the $H_{0}$ hypothesis if the *p*-value for any of the neighboring QTL pairs is smaller than α=0.05. The type I error for testing this single hypothesis is controlled to be smaller than α without corrections for multiple testing, for which empirical evidence is provided in a simulation study that is included in the Supplemental File S2.

### Web portal

To implement the meta-QTL hypothesis testing, we constructed an open source single page web application using HTML5 and JavaScript, hosted on GitHub pages (https://pages.github.com/). The source code is available at our GitHub “ramesh8v” repository (https://github.com/ramesh8v/tools). At the portal created from this source code (https://tools.omicsspeaks.com/consmap), a tab was built for each of the first three tests (QTL *vs.* QTL, QTL *vs.* Mendelian gene, and QTL *vs.* cluster mean). The portal provides a menu for selection of the *Ae. tauschii* chromosome and two input boxes to enter query and target locations in bp. The web application retrieves recombination rates for the query and target locations from the database of recombination rate grid points (Table S1). The mean of the retrieved recombination rates is computed and entered into the regression model to estimate the standard deviation, which together with the query and target locations is used to compute the *p*-value.

### Data availability

Table S1 contains the computed recombination rates at grid points on each of the seven *Ae. tauschii* pseudomolecules. Table S2 contains the roster and location of SSR markers projected onto the *Ae. tauschii* genome reference sequence. Table S3 contains the database of QTL projected onto the *Ae. tauschii* pseudomolecules. The projected markers and QTL were included into the Aet v4.0 JBrowse database. Supplemental File S1 provides information about JBrowse (http://aegilops.wheat.ucdavis.edu/jbrowse/index.html?data=Aet/data) databases and tracks containing 1,952 projected SSR markers, 284 projected SNP markers, and 585 projected QTL. Supplemental File S2 includes simulation testing the hypothesis that error rate is controlled to be smaller than α without corrections for multiple *p*-values. A link to the web portal for computing *p*-values for the three null hypotheses was included at the *Ae. tauschii* website hosting the JBrowse (above). All supplemental material (Tables S1, S2, and S3; Figures S1 and S2; and Files S1 and S2) are available at Figshare: https://doi.org/10.25387/g3.7607060.

## Results

### Projecting markers and QTL onto the Ae. tauschii pseudomolecules

Primers for 6,213 unique SSR markers were downloaded from the GrainGenes database. Of the 12,426 primers, 11,431 (92.0%) produced one or more hits in BLAST searches. A total of 907,476 hits with an average of 73 hits per primer were obtained. For 3,212 (51.6% of the initial number) primer pairs the hits were within 1 kb. The outcome did not appreciably change when the 1kb requirement was relaxed to 10 kb, in which case hits were within the prescribed interval for 3,277 primer pairs (52.7%). The set of 3,212 amplicons was used in BLAST searches against the *Ae. tauschii* genome sequence. Of them, 1,952 (31.4% of the initial number) were projected onto *Ae. tauschii* pseudomolecules (Table S2).

For other types of markers, only those that had been used for locating a QTL on a wheat genetic map were projected onto the *Ae. tauschii* pseudomolecules. A total of 284 SNP markers from the wheat 90K Infinium assay, 26 from the wheat 35K Infinium assay, and 61 from the wheat 9K Infinium assay, in addition to 34 SNP-containing GBS reads, two RFLP markers, four EST markers, and one SCAR marker were projected onto the *Ae. tauschii* genome sequence.

Data for 774 QTL mapped in wheat or wheat relatives were extracted from the literature, and 585 (75.6%) QTL for 154 traits were successfully projected onto the *Ae. tauschii* pseudomolecules (Table S3). Of 500 QTL originally mapped with SSR markers, 331 (66.2%) could be projected onto *Ae. tauschii* pseudomolecules, and of 258 QTL originally mapped with SNP markers 238 (92.2%) were successfully projected. Of the three wheat subgenomes the highest success rate with projecting QTL was with those in the D subgenome, 150/174 (86.2%), followed by those in the A subgenome, 168/232 (72.4%). The lowest success rate was with QTL in the B subgenome, 201/302 (66.5%).

Of the 585 QTL, 519 were discovered in purely wheat mapping populations whereas 66 QTL were discovered in wheat × wild relative mapping populations. Of the latter QTL, 30 involved *Ae. tauschii*, 33 involved wild emmer wheat, 2 involved *Triticum monococcum*, and 1 involved *Lophopyrum ponticum*. A total of 255 QTL were projected with two validated flanking markers and 330 were projected with a single validated marker. Of the latter, 24 QTL that were originally mapped relative to two flanking markers were anchored with only one validated flanking marker in this study, due to a failure to project the other marker onto the sequence. Fifteen QTL were projected onto the sequence with a single marker that could not be validated by any supporting marker and seven were projected by two adjacent markers on both sides of the single marker (Table S3).

The 585 QTL were classified into six groups based on the relationships among quantitative traits (Table 1). Most QTL belonged to trait group 3 (grain yield) followed by group 2 (biotic stress resistance) and group 4 (milling and baking quality).

**Table 1:** Distribution of projected QTL across the *Aegilops tauschii* genome

Pseudom.	Arm	Abiotic stress tolerance	Biotic stress resistance	Grain yield	Milling and baking quality	Morphology	Physiology	Total
1D	S	6	5	11	5	5	0	32
	L	4	6	8	10	3	2	33
	total	10	11	19	15	8	2	65
								
2D	S	28	15	16	9	6	11	85
	L	2	20	11	5	10	12	60
	total	30	35	27	14	16	22	145
								
3D	S	4	15	10	3	4	3	39
	L	6	10	6	5	13	8	48
	total	10	25	16	8	17	11	87
								
4D	S	15	2	9	7	12	2	47
	L	12	7	4	13	4	1	41
	total	27	9	13	20	16	3	88
								
5D	S	3	5	5	5	3	3	24
	L	10	15	13	8	8	17	71
	total	13	20	18	13	11	20	95
								
6D	S	3	4	11	4	0	0	22
	L	2	6	11	12	1	1	33
	total	5	10	22	16	1	1	55
								
7D	S	0	3	6	4	8	3	24
	L	0	4	17	9	2	2	34
	total	0	7	23	13	10	5	58
								
Whole genome		95	117	138	100	79	65	593

### Distribution of projected QTL in the Ae. tauschii genome sequence

The highest number of QTL was projected onto pseudomolecule 2D and the lowest onto pseudomolecule 6D (Table 1), which mirrored the relative sizes of these chromosomes in the *Ae. tauschii* genome, the largest and the smallest, respectively. Three QTL mapped onto wheat chromosome 5A were projected onto pseudomolecule 4D whereas two other QTL mapped on wheat chromosome 4A were projected onto pseudomolecule 5D. Two QTL with pairs of flanking markers were included into our database but not assigned to any pseudomolecule since one marker was mapped on pseudomolecule 4D and the other on pseudomolecule 5D. These apparently incongruous projections were caused by the known 4A/5A translocation (Devos *et al.* 1995; Dvorak *et al.* 2018b).

A slightly larger number of QTL (320) was projected onto the long arms than onto the short arms (273 QTL) of the pseudomolecules. Pseudomolecule 2D was exceptional by having a greater number of QTL on the short arm than on the long arm. The average distance between projected QTL varied among the pseudomolecules, ranging from 7.2 Mb on pseudomolecule 2D to 14.3 Mb on pseudomolecule 7D. Genome-wide, the distances between QTL averaged 9.9 Mb.

**Figure 1:**
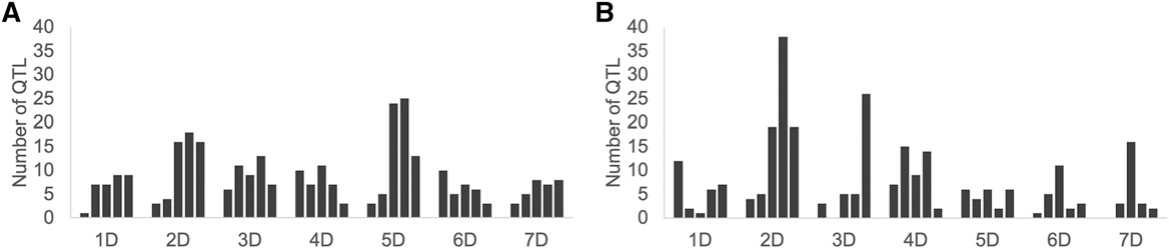
Distribution of QTL along the *Ae. tauschii* chromosome arms. The numbers of QTL (vertical axis) are expressed per bin each containing 20% genes in the arm. The bin closest to the centromere is to the left and that closest to the telomere is to the right in the histograms for the long (A) and short (B) arms of the seven *Ae. tauschii* chromosomes (horizontal axis).

To test the null hypothesis that every gene had the same probability to become a QTL, the genes in an arm were allocated into five bins containing equal number of genes, and QTL projected onto each bin were counted. If each gene had an equal chance to become a QTL, the numbers of projected QTL in the five bins would be equal. Only one of the 14 chromosome arms, 5DS, had similar numbers of QTL in all bins (Figure 1). In the remaining 13 chromosome arms the numbers of QTL increased in the proximal-to-distal direction or increased to about the midpoint and then declined toward the distal end. A similar distribution of QTL frequencies was found by using a non-overlapping window of 200 genes (Figure S1).

The null hypothesis that each gene in the genome had an equal chance to be mapped as a QTL was formally tested by applying a *χ^2^* test for the five bins. This gave *P* = 9 × 10^−6^ for the short arm and *P* = 1 × 10^−4^ for the long arm. Based on these low *p*-values, we concluded that genes along chromosome arms differ in the likelihood to be mapped as a QTL.

**Figure 2:**
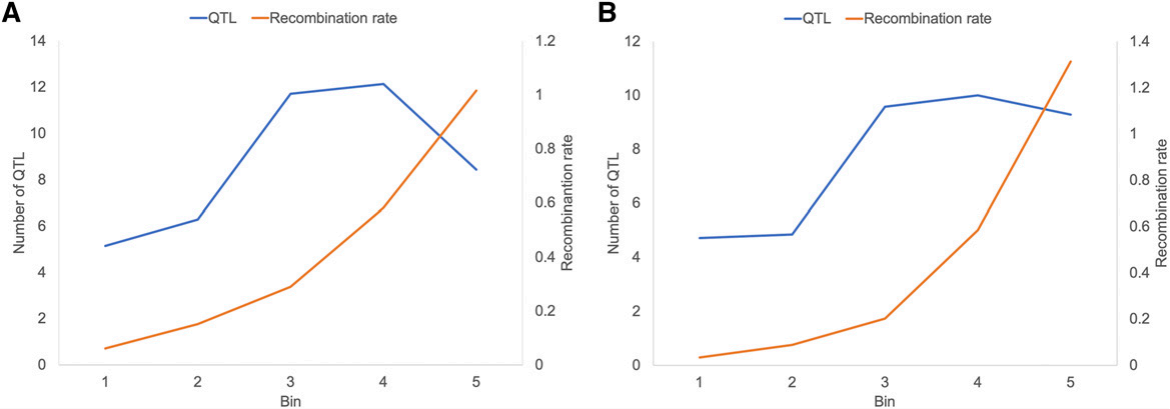
Relationship between frequencies of projected QTL, recombination rates, and location on chromosome arms. HC genes annotated in each *Ae. tauschii* chromosome arm were allocated into five bins, each containing 20% of the genes. The bins were arranged along the centromere-telomere axes. The centromere is to the left and the telomere is to the right. The numbers of QTL projected into each of the five bins were averaged across the seven chromosome arms (Figure 1) and plotted (blue lines). Recombination rates in cM/Mb in each of the five bins were averaged across the seven long arms (A) and seven short arms (B) and plotted (red lines).

Since the average frequency of QTL appeared to increase along chromosome arms (Figure 2), we investigated whether the probability of a gene to be mapped as a QTL was correlated with recombination rate in its vicinity. The linear correlation of the number of QTL per bin and mean recombination rate in the bin was computed (*r* = 0.28, *P* = 3 × 10^−4^, *N* = 158). We concluded that the probability for a gene to be mapped as a QTL was related to the recombination rate in its vicinity. However, the numbers of QTL declined (long arms) or plateaued (short arms) in the most distal bins while recombination rates increased. The distributions of QTL in the last bin was similar for QTL mapped with SNP markers and SSR markers (Figure S2).

### Meta-QTL hypothesis testing

Standard deviation estimates $\hat{\sigma }$ (in Mb) were obtained for eight clusters of QTL with their known causative Mendelian loci projected onto the *Ae. tauschii* genome sequence (Table 2). The clusters were located on five of the seven *Ae. tauschii* pseudomolecules and all involved QTL originally mapped on two or all three wheat homeologous chromosomes (for descriptions of the eight Mendelian loci and QTL see Methods). Several clusters involved QTL mapped in wild relatives of wheat. The estimates of $\hat{\sigma }$ greatly varied, ranging from 0.6 Mb to 43 Mb among the eight clusters (Table 2).

**Table 2:** Clusters of QTL used for estimating standard deviation

Cluster	Trait	QTL_ID	Mapped on	Projected to	Location (bp)	Marker type	Mendelian gene (ID no.)^1^	Start on pseudom. (bp)	$\hat{\sigma }$(Mb)
1	GYTKW	Q6195	6A	6D	153,709,664	SSR	*GW2* (4328856)	200,039,189	31.6
		Q6205	6B	6D	213,676,781	SNP			
		Q6295	6A	6D	228,005,026	SNP			
									
2	MBQ_SED	Q1411.1	1D	1D	419,300,752	SSR	*Glu-1* (105262590)	419,364,660	34.9
	MBQ_DS	Q1411.2	1D	1D	419,300,752	SSR			
	MBQ_DS	Q1417	1B	1D	421,556,928	SSR			
	MBQ	Q1445	1B	1D	454,167,040	SSR			
	MBQ_PH	Q1450.2	1B	1D	462,917,844	SSR			
	MBQ_TE	Q1450.3	1B	1D	462,917,844	SSR			
	MBQ_BWE	Q1450.1	1B	1D	462,917,844	SSR			
	MBQ_TW	Q1450.4	1D	1D	462,917,844	SSR			
	MBQ_GPC	Q1460	1B	1D	484,762,306	SNP			
	MBQ_FN	Q1555	1B	1D	500,605,788	SSR			
									
3	MBQ_SED	Q1005	1A	1D	5,897,318	SNP	*GLEN42K* (542805)	5,072,910	3.5
	MBQ_TW	Q1033	1B	1D	11,688,104	SSR			
	MBQ_GH	Q1050	1A	1D	12,255,558	SSR			
	MBQ_SED	Q1065	1D	1D	13,539,062	SNP			
	MBQ_TW	Q1080	1A	1D	36,662,483	SNP			
									
4	VR	Q5422	5A	5D	425,007,359	SSR	*Vrn-B1* (543103)	476,114,154	43.0
		Q5458	5A	5D	462,769,675	SSR			
		Q5491	5B	5D	485,754,518	SSR			
		Q5532	5A	5D	512,106,475	SSR			
									
5	SRR	Q1013	1DS	1D	9,511,649	SNP	*Yr10* (542843)	1,911,967	0.6
		Q1020	1DS	1D	9,789,725	SNP			
		Q1058	1BS	1D	12,269,471	SNP			
									
6	SKC	Q2555.5	2A	2D	563,628,397	SNP	*HKT4* (109755894)	538,975,581	5.7
		Q2568.2	2A	2D	570,054,415	SSR			
									
7	HD	Q2034.7	2DS	2D	20,395,218	SSR	*Ppd-1* (109761523)	35,205,016	16.8
		Q2034.5	2D	2D	20,395,218	SSR			
	DTM	Q2047.1	2D	2D	24,288,494	SSR			
	EET	Q2067.2	2D	2D	49,117,097	SSR			
	HD/PS	Q2073	2D	2D	53,493,220	SSR			
	FT/PS/IE	Q2084	2B	2D	61,502,257	SSR			
									
8	PTH	Q4050	4BS	4D	22,922,715	SNP	*Rht-1* (109733020)	20,534,075	16.1
		Q4055	4DS	4D	24,896,742	SNP			
		Q4077.2	4D	4D	39,049,282	SSR			

1 Gene identification number in the NCBI gene database.

**Figure 3:**
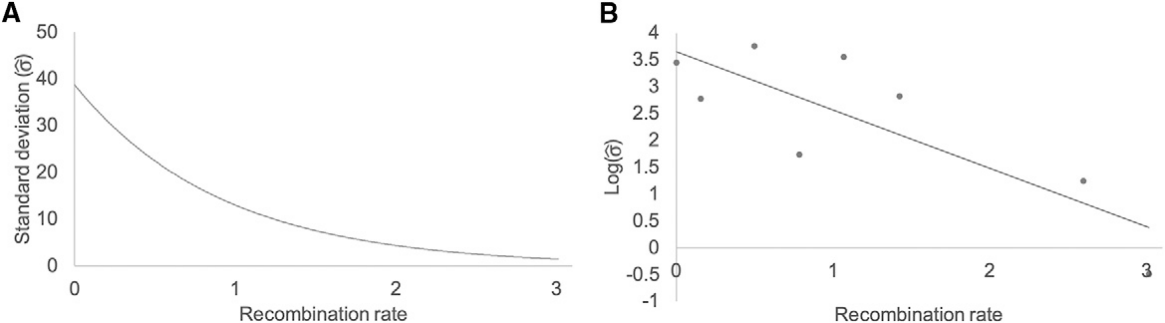
Relationship between recombination rate (Rt) and the size of standard deviation ($\hat{\sigma }$). (A) The standard deviation of independently mapped QTL for a Mendelian locus ($\hat{\sigma }$) in Mb is on the vertical axis and the mean meiotic recombination rates (Rt) in cM/Mb in the vicinity of the projected QTL within a cluster are on the horizontal axis. The size of $\hat{\sigma }$ exponentially decreases as Rt increases. (B) The relationship between log($\hat{\sigma }$) and Rt is linear.

 The local density of genes and SSR markers along the *Ae. tauschii* chromosome arms (Luo *et al.* 2017) and the local density of markers on genetic maps in Triticeae correlate with recombination rate along chromosome arms. We therefore hypothesized that the sizes of deviations of independent attempts to map a QTL with different markers on different maps would be larger for QTL proximally located than for those distally located and would be related to the local recombination rate. The relationship between $\hat{\sigma }$ and Rt was fitted by an exponential relationship (Figure 3A), for which the log response was linearly related to the predictor (Figure 3B). The variable $\log\;\left(\hat{\sigma }\right)$ was related to Rt by the linear regression equation

$$\log\left(\hat{\sigma }\right)=\text{α}+\beta \text{Rt}+\epsilon .$$

The intercept α and slope β were estimated as $\hat{\alpha }=3.656$ and $\hat{\beta }=-1.089$ (*P* = 0.006, one-sided *t*-test) for the regression effect, rejecting the null hypothesis of β=0 for the regression line (Figure 3B) in favor of a negative slope of the regression line and a decaying exponential relationship of the standard estimation with Rt, which confirmed the above-mentioned relationship. These parameter fits and Rt along the *Ae. tauschii* chromosomes can be used to compute $\hat{\sigma }$ adjusted for recombination rate and the estimates can then be used for conducting the first three hypothesis tests described in Methods.

To assess the effect of marker type on the sizes of standard deviations estimated by the statistical model, a *t*-test based on adjusted distances of QTL mapped by SSR and SNP markers was performed for the null hypothesis *H_0_*: adjusted deviations of QTL mapped by two different marker types are the same. The mean adjusted distance between QTL mapped with SNP markers was 1.68 Mb and that of QTL mapped with SSR markers was 1.54 Mb. The test yielded *P* = 0.846. We therefore could not reject the null hypothesis and concluded that there was no evidence that our model would not be applicable to QTL mapped by either type of markers.

### Web portal for hypothesis testing

A web portal (https://tools.omicsspeaks.com/consmap) was constructed to facilitate the computation of $\hat{\sigma }$ adjusted for recombination rate and *p*-values for the three hypotheses about QTL projected onto the *Ae. tauschii* genome sequence. The portal contains tabs, each of which implements one of the hypothesis tests described in Methods. The tab “Test1: QTL vs QTL” was designed for testing the null hypothesis that two QTL are independent estimates of a single locus. The tab “Test2: QTL vs Mendelian locus” was designed for testing the null hypothesis that a QTL is an estimate of variation in QTL mapping of a specific locus annotated on or projected onto the Aet v4.0 sequence. The tab “Test3: QTL vs cluster mean” was designed to test the null hypothesis that a projected QTL is a member of an existing QTL cluster. QTL cluster should be based on biological evidence that the QTL are independent estimates of the same locus, albeit of unspecified location on an Aet v4.0 pseudomolecule. Each *Ae. tauschii* chromosome has a slightly different distribution of recombination rates, which necessitates inputting the respective *Ae. tauschii* chromosome. Next, the user should input the pseudomolecule coordinates of the projected query QTL. Finally, the user should input the pseudomolecule coordinates of the projected target QTL for Test 1, a coordinate for the Mendelian locus for Test 2, and the coordinates of all QTL in the cluster for Test 3. A *p*-value for the requested test is then computed.

### A case study

We used QTL for resistance to *Fusarium* head bight (FHB), also known as scab, to illustrate the utility of projecting QTL on the *Ae. tauschii* genome sequence and the use of our Web portal for meta-QTL hypothesis testing. A total of 64 QTL for FHB resistance was projected onto the *Ae. tauschii* pseudomolecules (Tables 3 and S3). A QTL mapped on 7E chromosome of *Thinopyrum ponticum* was not considered. The largest number of them were on chromosomes in the homeologous group 3. In total, 3, 16, and 2 QTL were on chromosomes 3A, 3B, and 3D, respectively. The disproportionally greater number of QTL on 3B was due to numerous attempts to map the *Fhb1* gene, which provides the most effective resistance to FHB in wheat. The gene is in the distal region of 3BS (Waldron *et al.* 1999).

**Table 3:** Minimum number of genes affecting *Fusarium* head blight resistance in wheat and projected onto the *Ae. tauschii* genome sequence

Pseudomolecule	QTL no.	Minimum no. of genes
1D	2	2
2D	17	4
3D	21	3
4D	7	2
5D	11	3
6D	4	2
7D	2	2
Total	64	18

**Figure 4:**
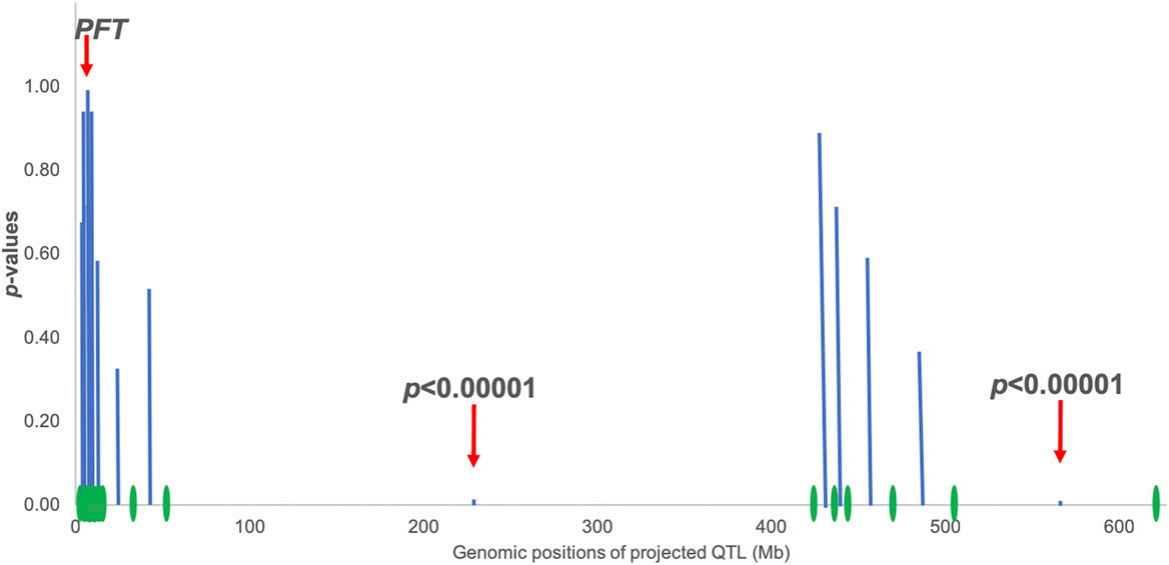
Tests of the null hypothesis that neighboring FHB QTL correspond to the same gene. The tests involved 21 QTL for various phenotypes of resistance to *Fusarium* head blight (FHB) mapped on wheat chromosomes 3A, 3B, and 3D and individually projected onto pseudomolecule 3D. The QTL *vs.* QTL option implemented in the Web portal was used to compute the *p*-values for testing whether each pair of neighboring QTL were from the same cluster. The approximate projections of QTL (green ovals) on pseudomolecule 3D are indicated on the horizontal axis. Computed *p*-values (blue vertical columns) are on the vertical axis. The null hypothesis was rejected for two pairs of neighboring QTL (red arrows): Q3088-Q3427 and Q3505-Q3695 (Table S3) but could not be rejected for the remaining intervals between neighboring QTL. The location of *PFT*, the causative gene for *Fhb1* (Rawat *et al.* 2016) is indicated.

A meta-QTL analysis of the 21 QTL on chromosomes 3A, 3B, and 3D was conducted to assess the minimum number of genes on these three chromosomes and to find if some QTL are duplicated on homeologous chromosomes. Using the QTL *vs.* QTL hypothesis testing option in the portal, *p*-values between neighboring QTL were computed (Tests 1, Methods). The resulting *p*-values were above 0.05, except for intervals Q3088-Q3427 and Q3505-Q3695 for which the *p*-values were <0.0001 (Figure 4). Based on these *p*-values we rejected the hypothesis that all 21 QTL form a single cluster, leading to the conclusion that there are at least three different genes among wheat chromosomes 3A, 3B and 3D affecting FHB resistance (Test 4, Methods). We should emphasize that failing to reject the null hypothesis *H_0_* for a group of neighboring QTL with *p*-values > 0.05 would not warrant the statement that these QTL form a cluster equivalent to a single Mendelian gene, as this would require an additional equivalence test in order to accept the null hypothesis with confidence. Hence, only the minimal but not the exact number of FHB genes needed to account for the QTL clusters can be determined.

We then used Test 4 (Methods) to estimate the minimum number of genes underpinning the set of 64 FHB QTL. We estimated that a minimum of 18 different genes for FHB have been detected in wheat (Table 3).

*Fhb1* locus was isolated and shown to the equivalent to gene *PFT* encoding a wheat agglutinin protein (Rawat *et al.* 2016). Blast with the *PFT* gene (AOZ21512.1) against the AL8/78 genome sequence detected a locus on 2D at 634,015,182 to 634,016,319 bp (annotated as AET2Gv21236400 (http://aegilops.wheat.ucdavis.edu/ATGSP/index.php). The gene was 88% identical with *PFT* indicating that it was a paralogue of *PFT*. No locus was detected on 3D. To predict an approximate location of the *PFT* in the Aet v4.0 genome sequence, we projected gene AOZ21513.1, a neighbor of *PFT* on 3B (Rawat *et al.* 2016), onto the *Ae. tauschii* genome sequence. The gene was located at 7,382,740 bp on the 3D pseudomolecule.

Since no gene corresponding to *PFT* was on 3D, it was unexpected to find QTL QFhs.fal-3DS reported on 3DS (Paillard *et al.* 2004). The 3D QTL (Q3014) was projected at 4,887,606 bp. To test the hypothesis that QFhs.fal-3DS is a QTL of a *PFT* ortholog on wheat 3D, the “QTL vs Mendelian gene” option in the Web portal (Test 2, Methods) was used (*P =* 0.53). Based on this *p* value we could not reject the hull hypothesis. It is therefore possible that chromosome 3D in the Arina × Forno RIL population (Paillard *et al.* 2004) segregated for an ortholog of *PFT*.

Another biological question that emerged from the meta-QTL analysis was whether QFhs.nau-2DL (Jiang *et al.* 2007) projected on 2D at 522,697,513 could be a QTL of the AET2Gv21236400 locus on 2D. The QTL *vs.* Mendelian gene option in the portal (Test 2, Methods) yielded *P* < 0.0001. We therefore rejected the null hypothesis of equivalence of the FHB resistance QTL QFhs.nau-2DL on 2D and the expression of the AET2Gv21236400 agglutinin gene.

## Discussion

### Markers

Of the 6,385 random wheat SSR markers downloaded from GrainGenes, only 30.6% were successfully projected onto the *Ae. tauschii* genome sequence. This success rate was lower than the 83.6% obtained with projecting A- and B-subgenome SNP markers mapped in the wild emmer onto the *Ae. tauschii* genome sequence (Jorgensen *et al.* 2017). A likely reason for this great difference was subgenome specificity, which was emphasized in the design of SSR markers (Röder *et al.* 1998) but not in the design of SNP markers for Illumina assays (Akhunov *et al.* 2009).

Projecting QTL mapped with SSR markers was more successful, about 66.2%, which was primarily due to that it was sufficient to project only one of two SSR markers that were used to map a QTL on a genetic map. A marker that failed to be projected was often replaced by another SSR marker mapped in its vicinity, provided that the alternative marker survived the validation steps described in Methods.

The greater success rate of projecting QTL mapped with SNP markers, 92.2%, than with SSR markers indicated that meta-analyses and discovery of candidate genes will likely be more successful for QTL mapped with SNP markers than for those mapped with SSR markers. If possible, it is therefore preferable to use SNP markers rather than SSR markers in QTL discovery and mapping in wheat and its relatives.

### QTL distribution along chromosomes

A total of 585 of the 774 QTL accessed in the literature were projected onto the *Ae. tauschii* pseudomolecules. The projections of this large number of QTL onto a single genomic reference made it possible to test the hypothesis that the QTL distribution was the same as the distribution of genes along the centromere-telomere axes of wheat chromosome arms. This hypothesis was rejected with very low *p* values for both short and long arms. Compared to gene distribution, QTL distribution increased in the centromere-to-telomere direction, peaking in the third or fourth bin and then plateaued or declined in the fifth bin. To account for this pattern, we considered the possibility that QTL locations were often equated with midpoints between flanking markers, which may have shifted the locations of QTL away from the telomeric ends. We hypothesized that if this were true, QTL mapped with SNP markers would show more even distribution among the five bins than QTL mapped with SSR markers because SNP maps are usually more marker dense than SSR maps, and the distances between markers flanking LOD maxima are often shorter on SNP maps than on SSR maps. However, our comparisons of the distribution of SNP-mapped QTL with the distribution of SSR-mapped QTL showed no meaningful difference.

Except for the anomalous distribution of QTL in the distal portions of chromosome arms, the distribution of QTL along the rest of the chromosome arms mirrored recombination rates along chromosome arms and correlated with them. This is perhaps not surprising since QTL are a form of genetic diversity in the genome and genetic diversity correlates with recombination rate in wheat and its relatives (Dvorak *et al.* 1998; Akhunov *et al.* 2010; Wang *et al.* 2013). Furthermore, gene duplications and deletion, which contribute to the evolution of genetic novelty in wheat and its diploid relatives also correlate with recombination rates (Dvorak and Akhunov 2005). Disease resistance genes, which represented a large portion of QTL in our study, are preferentially located in high-recombination regions in the *Ae. tauschii* genome (Luo *et al.* 2017) and likely also in the wheat genome.

### Meta-QTL analysis

Standard deviations of QTL projections on the *Ae. tauschii* genome sequence varied from 0.6 to 43 Mb among the eight QTL clusters in Table 2, which was mostly caused by variation in recombination rates along chromosomes. There may be several reasons for the relationship between $\hat{\sigma }$ and recombination rate. Gene density is highest in distal, high-recombination regions of wheat and *Aegilops* chromosomes (Akhunov *et al.* 2003; Luo *et al.* 2017). The density of markers within or near genes, the preferred locations of most of the wheat SSR and SNP markers, will therefore also be highest in the distal, high-recombination regions. Physical distances between neighboring markers on a genetic map are therefore shorter in the distal, high-recombination chromosome regions than in the proximal, low-recombination chromosome regions. The deviations of projected QTL from the mean QTL location on the pseudomolecule will therefore be smaller in high-recombination regions than in low-recombination regions and the size of $\hat{\sigma }$ of QTL mapping and projection will be related to local recombination rate. The same relationship is also expected due to the distortion of genetic maps caused by preference of crossovers for distal chromosome regions over the proximal chromosome regions in wheat and its relatives.

Based on the exponential decay relationship between $\hat{\sigma }$ and recombination rate, we developed a model for adjusting $\hat{\sigma }$ for local recombination rate and deployed it in a portal with a graphical user interfase (GUI) for testing hypotheses about a QTL projected onto an *Ae. tauschii* pseudomolecule. The model and its application assume that no other factor affects the sizes of deviations among independently mapped and projected QTL. For example, we made no distinction between QTL mapped with SSR and SNP markers in the model. If $\hat{\sigma }$ were actually smaller for QTL mapped with SNP markers than for those mapped with SSR markers, the model would overestimate the *p*-value for QTL mapped with SNP markers and underestimate that for QTL mapped with SSR markers. Our test of this hypothesis failed to find a difference between QTL mapped with SSRs and SNPs. However, that does not mean that this will continue to be true. Improvements in mapping technology may necessitate re-evaluation of the model and adapting it to the increased precision of QTL mapping. Another factor that the model does not take into account is the size of the sample of gametes that were employed in QTL mapping. If sample size and type of mapping population, *e.g.*, F_2_ compared to recombinant inbred lines, would systematically affect the size of deviations, the model would again be unrealistic. As more data become available, it will be possible to evaluate the various variables affecting the precision of QTL mapping and modify the model accordingly.

To facilitate meta-analyses of QTL projected onto the *Ae. tauschii* genome sequence, we constructed a portal for testing hypotheses about a projected query QTL, namely, whether it may be an estimate of another projected QTL (Test 1), whether it may be an estimate of a specific Mendelian gene (Test 2), or whether it may belong to a QTL cluster (Test 3). The test of whether a set of QTL belong to the same cluster (Test 4) can be constructed from consecutive applications of Test 1 and requires no multiple-test adjustments as demonstrated by a simulation (Supplemental File S2). The rejection of $H_{0}$ in Test 4 provides statistical evidence for the existence of multiple clusters but not the number of clusters. It should also be emphasized that the failure to reject the null hypothesis for a group of tests of neighboring QTL should not be interpreted as evidence that all such neighboring QTL belong to a single locus. Thus, the 13 QTL surrounding the *Fhb1* locus and projected onto the tip of the short arm of the 3D pseudomolecule spanning an interval 4,887,606 to 50,957,578 bp in our case study may not all be QTL of *Fhb1*, even though all of the tests of neighboring QTL failed to reject the null hypothesis.

In our analyses of the eight QTL clusters used for estimating $\hat{\sigma }$ and the FHB QTL in the case study, we conducted meta-analyses only with QTL for related traits. The tools we developed and made available here could also be used for meta-analyses between collocated but superficially unrelated traits. Such QTL collocations may arise from pleiotropy or tight linkage of causal genes. The former was suggested to account for the collocation of QTL for several traits with a major QTL for spike shattering on the long arm of wild emmer chromosome 2A (Peng *et al.* 2003; Peng *et al.* 2013). Similar collocated QTL for superficially unrelated morphological traits were encountered on other wheat chromosomes and also considered to be pleiotropy (Peng *et al.* 2003; Peng *et al.* 2013). Other studies of the genetic bases of wheat domestication added additional QTL to the collocated QTL reported by Peng *et al.* (Tzarfati *et al.* 2014; Thanh *et al.* 2013; Peleg *et al.* 2011). Projecting QTL on the *Ae. tauschii* genome sequences or wild emmer genome sequence now available (Avni *et al.* 2017) will greatly improve the use of high-resolution genetic mapping and facilitates reverse genetic approaches to decide whether pleiotropy or tight linkage is responsible for specific cases of QTL collocation in wild emmer × domesticated wheat and similar QTL collocations in other wheat QTL mapping populations.

The database of projected QTL on the *Ae. tauschii* genome sequence is an open-ended resource to which new QTL can be easily added using BLASTN of marker sequences against the *Ae. tauschii* Aet v4.0 genome sequence (http://aegilops.wheat.ucdavis.edu/ATGSP/blast.php) to find pseudomolecule coordinates for new QTL. The coordinates can then be used to compare new QTL with already discovered QTL using the portal or search for genes identified in other grass genomes using collinearity databases for the *Ae. tauschii* genome (Luo *et al.* 2017; Dvorak *et al.* 2018a).
